# Melt-driven erosion in microparticle impact

**DOI:** 10.1038/s41467-018-07509-y

**Published:** 2018-11-29

**Authors:** Mostafa Hassani-Gangaraj, David Veysset, Keith A. Nelson, Christopher A. Schuh

**Affiliations:** 1 Department of Materials Science and Engineering, MIT, Cambridge, MA 02139 USA; 2 Institute for Soldier Nanotechnologies, MIT, Cambridge, MA 02139 USA; 3 Department of Chemistry, MIT, Cambridge, MA 02139 USA

## Abstract

Impact-induced erosion is the ablation of matter caused by being physically struck by another object. While this phenomenon is known, it is empirically challenging to study mechanistically because of the short timescales and small length scales involved. Here, we resolve supersonic impact erosion in situ with micrometer- and nanosecond-level spatiotemporal resolution. We show, in real time, how metallic microparticles (~10-μm) cross from the regimes of rebound and bonding to the more extreme regime that involves erosion. We find that erosion in normal impact of ductile metallic materials is melt-driven, and establish a mechanistic framework to predict the erosion velocity.

## Abstract (web summary)

Supersonic particle impacts can cause permanent damage to space vehicles and satellites, but how exactly remains unclear. Here, the authors visualise for the first time the high impact of single tin microparticles on a tin substrate and show erosion of ductile metallic materials is melt-driven.

## Introduction

The combination of high pressures, temperatures, and deformation rates that occur during an impact can evoke many unusual materials responses. These include crater formation^1–3^, solid state splashing^4^, impact bonding^5^, peculiar phase transformations^6^, nanocrystallization^7^, and chemical reactions^8^. Among the most extreme of such phenomena lies impact-induced erosion that often occurs at the micron scale whether in space^9^, on the ground^10^, or beneath^11^. At the micron scale, metallic microparticles change their interaction with metallic targets as impact velocity increases. In the limit of extremely weak impact (<~0.1 m/s), an elastic response occurs; the particles rebound with their initial kinetic energy recovered^12^. At higher impact velocities (~10 m/s), impacting microparticles bounce off with a fraction of their initial kinetic energy while plastically deforming themselves and the target^13^. At even higher impact velocities (~100 m/s) the incoming microparticles can adhesively bond to the target (substrate in this case) and additively build-up coatings or bulk components^14–16^. In neither of these first regimes, i.e., rebound nor bonding, is material lost from the microparticle or the substrate. At higher impact velocities in the third regime, on the other hand, material loss can occur; velocities of ~km/s cause impact damage to, for instance, space vehicles and satellites^9,17,18^. However, such high-velocity erosion has been historically studied by post-mortem analysis only^19,20^. Without direct observation, there remain many fundamental questions pertaining to the mechanisms of impact-induced erosion, and conditions required to even trigger erosion are not resolved.

Here, we conduct in situ single microparticle supersonic impacts aimed at systematically elucidating erosion with micron scale and nanosecond level spatiotemporal resolution. Furthermore, we establish a mechanistic framework to predict the erosion velocity.

## Results

### In situ and post-mortem measurements

**Fig. 1:**
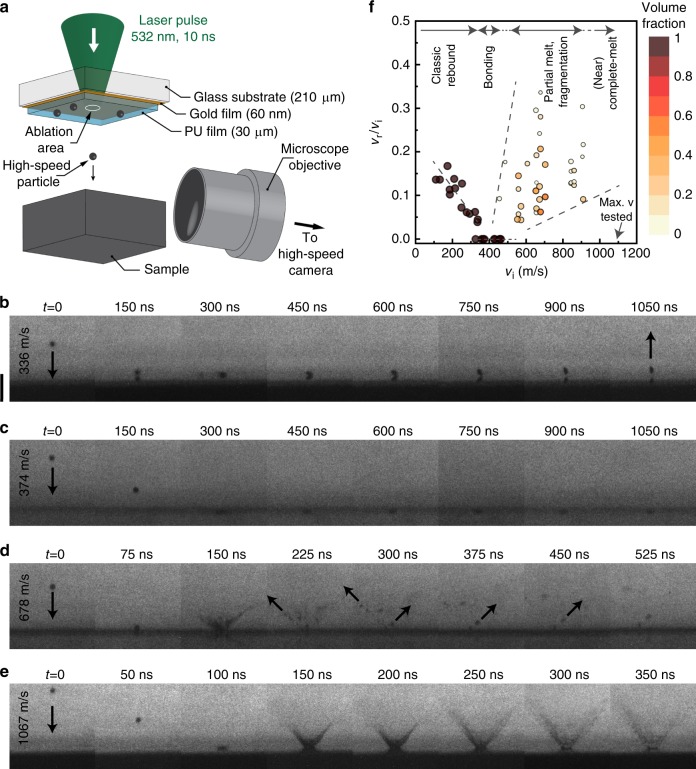
In situ observation of microparticle supersonic impact. **a** Experimental platform for microparticle impact test and real-time imaging. **b**–**e** Multi–frame sequences with 5 ns exposure times showing (**b**) 9 µm, (**c**) 9 µm, (**d**) 10 µm, and (**e**) 10 µm tin particles arriving from the top of the field of view and impacting tin substrates at velocities of (**b**) 336, (**c**) 374, (**d**) 678, and (**e**) 1067 m/s, spanning from the rebound regime to the bonding and the erosion regimes. The scale bar is 50 μm. (**f**) Coefficient of restitution, *v*_r_/*v*_i_, of the rebounding tin particles and fragments. The coefficient of restitution is equal to zero in the bonding regime, and it is non-zero in the rebound and erosion regimes. The size of the data points on the plot is proportional to the ratio of the fragment and particle diameters. We also color-coded volume fraction, i.e., volume of the rebounding particle/fragment divided by the volume of the incoming particle

We choose tin as a model material for its low melting temperature and specific heat. ﻿As schematically shown in Fig. 1a, micron-sized particles are initially dispersed on a launching pad assembly, a stack comprising a glass substrate, a thin layer of gold, and a layer of elastomeric polyurea film. Microparticles are launched by focusing a laser excitation pulse, thereby ablating the gold film, and causing rapid expansion of the polyurea film^4,5,21,22^. The particle interaction with a substrate is recorded in real time using a high-frame-rate camera and a synchronized quasi-cw laser imaging pulse for illumination. The exposure was set to be 5 ns for all frames, and the interframe time was adjusted depending on the impact velocity from 50 to 150 ns. Details regarding the launching pad preparation are described elsewhere^4,5,23^.

Figure 1b–e show some exemplar snapshots capturing tin microparticles impacting tin substrates. We explored the interaction between particle and substrate at various impact velocities, from 100 to 1100 m/s, and observed four distinct behaviors. First, at low impact velocity, the particle bounces off the substrate as shown in Fig. 1b (336 m/s). Second, with increasing impact velocity (374 m/s), the particle in Fig. 1c no longer rebounds, but instead bonds to the substrate adhesively. Third, increasing the impact velocity further to 678 m/s leads to a material splash involving ejected fragmented pieces of material (Fig. 1d). Fourth, at the high-end of our velocity scale at 1067 m/s we observe only a stark splash, with no discernible individual fragments in the cloud of ejecta (Fig. 1e). These image sequences have been montaged into Supplementary Movies 1-4 and are available in the Supplementary Information.

**Fig. 2:**
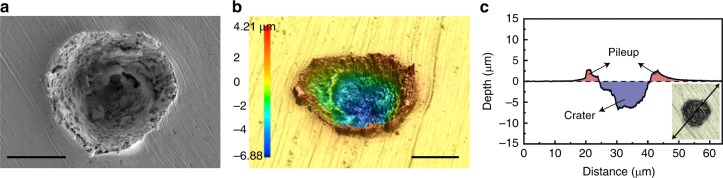
Surface profilometry of a typical impact in the erosion regime. (**a**) Scanning electron micrograph, (**b**) laser confocal image of the impact area, and (**c**) a typical surface profile after a 10-µm tin particle impacted the tin substrate at 1036 m/s velocity. The color bar in (**b**) represents surface height. While the apparent crater volume is 742 μm^3^, the pileup volume is 626 μm^3^ confirming that at least a volume of 116 μm^3^ is lost from the substrate during impact. The scale bars are 10 μm

We measured the impact velocity, rebound velocity, size of the impacting particle, and size of the rebounding particle (or fragments of it) for 68 experiments. Figure 1f shows how the coefficient of restitution (the ratio of rebound, *v*_r_, and impact, *v*_i_, velocities), varies with impact velocity. We observe an apparent linear decrease in *v*_r_/*v*_i_ for the impact velocity range of 100–350 m/s, which is the regime where the particle bounces. The sharp decrease in this ratio to zero at ~350 m/s identifies the transition from rebound to bonding. In our previous work we have measured this critical velocity for bonding for a number of metals^4^. New to the present work is the observation of a second transition at around 450 m/s, to a third regime of behavior where there is material ejection from the impact with non-zero *v*_r_/*v*_i_ values; the quantitative values reported in Fig. 1f correspond to the ejection velocities of the small fragments that could be discerned in our photographs. As splash and fragmentation lead to material loss, we refer to this regime as the erosion regime. A scanning electron micrograph of a crater left behind after an impact in the erosion regime, as well as its reconstruction using 3D surface profilometry, are shown in Fig. 2a, b respectively. Crater and pileup can be observed in Fig. 2c in a profile across the line shown in the inset. An impact involving particle deposition would create more pileup volume than crater volume. By contrast, our analysis of Fig. 2b shows that the crater volume is 116 μm^3^ larger than the pileup volume. Considering that there may still be some material deposition from the particle, our analysis confirms that at least a material volume of 116 μm^3^ is lost from the substrate.

Another interesting feature of Fig. 1f is that the erosion regime involves a wide range of ejection velocities, owing to the ejecta comprising a variety of small fragments. We have elected to scale the size of particles/ejecta with the size of the data points and represent the volume fraction (of the ejecta of each size) with the color scale in Fig. 1f, which helps visualize the distribution of different particles and their velocities during the erosion event. In general, the larger volumes of ejecta tend to travel more slowly. What is more, it is possible to distinguish a further, more subtle transition within the erosion regime. In the first sub-regime, from ~450 to ~900 m/s, solid fragments are easily discerned and significantly contribute to the total material loss. As impact velocity increases, fragment sizes tend to decrease to the point where no significant individual fragment can be observed. For any impact velocity higher than ~900 m/s we observe that material is almost entirely lost in an ejecta cloud which we interpret to be liquid splashing, as further developed below.

**Fig. 3:**
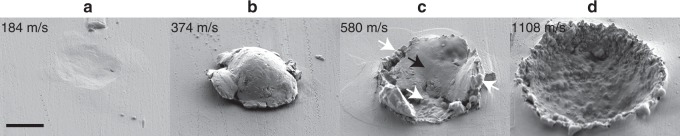
Post-mortem observations of impact area. Scanning electron micrographs of the impact areas after (**a**) 9 µm, (**b**) 9 µm, (**c**) 10 µm, (**d**) 10 µm tin particles impacted the tin substrate at (**a**) 184, (**b**) 374, (**c**) 580, and (**d**) 1108 m/s velocities, showing (**a**) rebound, (**b**) bonding, (**c**) partial melt, and (**d**) full melt. Corresponding in situ observations confirm material loss in (**c**) and (**d**). The scale bar is 5 μm

Post-mortem observations of the impact sites confirm these four behavioral regimes identified in situ above. In the rebound regime, an indentation such as shown in Fig. 3a is left behind on the substrate. Particles exceeding the critical bonding velocity, such as the one in Fig. 3b, adhere to the substrate without substantial material loss. At higher velocities where erosion sets on, impacts leave residue behind. Figure 3c at 580 m/s exhibits two different morphologies, one indicated by black arrows that appears formed by solid plasticity, and a second indicated by white arrows that is rounded and smooth and suggestive of melting and resolidification. This type of residue is consistent with our in situ observations in that some large solid fragments might be seen to form from plastic deformation and fracture, while a fine spray of ejecta could be a result of impact melting and splashing. Finally, in the highest speed regime, where we exclusively see splashes without discernible fragments, the impact site appears to be a completely melted and resolidified crater, as shown in Fig. 3d. Although we cannot directly discern melting in the substrate from the post-mortem observations in Fig. 3c, d, we note that both particle and substrate have the same material properties and undergo a conformal plastic deformation as confirmed by the surface profile in Fig. 2c. Therefore, melting in the particle can be a reasonable indictor of melting in the substrate.

### Erosion mechanism

A critical conclusion that emerges by combining our postmortem and in situ observations is that significant material loss and melting emerge concurrently at the transition velocity between the bonding and the erosion regimes. In other words, erosion appears to be melt-driven in the present case. This is mechanistically unforeseen based on prior work on erosion, although the significance of plasticity-induced heating and melting upon impact, especially at velocities on the order of 1 km/s has been pointed out recently^24,25^. The conventional wisdom about impact erosion of ductile metallic materials is based upon eroding metallic surfaces by a large number of particle impacts in a fluid stream^26–31^. In such conditions, material loss is mechanistically attributed to either a cutting action^11,32^ or a combined forging–extrusion action^33–35^ of the eroding particles. In the former, which is mostly relevant to oblique impacts, particles penetrate the substrate, translate along the surface and plow material ahead of them. In the latter, which is more relevant to normal-incidence impacts, small highly distressed platelets of the substrate material are envisioned to result at a first impact site, which can then be flaked off the surface by a subsequent impact. The cutting mechanism cannot be the predominant factor for material loss in the present normal-incidence impacts, and there is no succeeding particle to remove platelets formed by previous impacts as envisioned with the forging–extrusion mechanism.

Thus, we see our observation of melt-driven erosion as a departure from previously established erosion mechanisms. This is reasonable in light of two significant distinctions between our work and the studies of impact erosion cited above. First, our impact velocities are higher by a large factor, up to an order of magnitude with respect to some studies. Second, most studies employ particles significantly harder than the substrate they are eroding, while we have used a matched pair. Delineating property and velocity regimes in which different mechanisms are dominant may become possible if quantitative mechanistic models are developed; we pursue this in what follows for melt-based erosion.

## Discussion

As a starting point we take our experimental observation that melting and erosion appear to set on together at a single critical velocity in tin; predicting erosion thus hinges on assessing when an impact can trigger melting. In what follows we develop a first-order analysis in the spirit of prior analogous works from other domains^8,36^, so that we can map the most critical physics of the process across many possible materials. During a normal-incidence impact of a microparticle, the plastic work dissipates as heat, and in turn only a fraction of the kinetic energy of the impacting particle causes plastic work. At the onset of melt-driven erosion we can balance the input kinetic energy thusly dissipated with the energy required to heat and melt an affected volume of material:

1 $$\beta \times \alpha \times \frac{1}{2}m_{\mathrm {p}}v_{{\mathrm {i,e}}}^2 = \rho V_{{\mathrm {aff}}}\left[ {C_{p}(T_{\mathrm {m}} - T_0) + H_{\mathrm {f}}} \right]$$

where *β* is the fraction of plastic work dissipated as heat, *α* is the fraction of the initial kinetic energy of the particle deposited into the substrate, *m*_p_ is the mass of the particle, *v*_i,e_ is the impact velocity at the erosion onset, *ρ* is the density, *V*_aff_ is the affected volume over which the temperature rises from its initial value *T*_0_ to the melting temperature *T*_m_, *C*_*p*_ is the specific heat, and *H*_f_ is the enthalpy of fusion. For a first-order approximation, we assume that temperature is uniformly distributed in the affected volume. For simplicity we can take the affected volume as the product of the contact area of the impact, *A*_c_, and the distance, *d*_th_, heat is conducted during the contact time, $t_{\mathrm {c}} \approx d/v_{{\mathrm {i,e}}}$, where *d* is the particle diameter assuming that the affected volume can approximate the volume in which the inelastic heat is generated. We calculate the thermal distance using $d_{{\mathrm {th}}} = \sqrt {\left( {\frac{k}{{\rho C_{\mathrm {p}}}}} \right) \times t_{\mathrm {c}}}$ with *k* being the thermal conductivity. In order to estimate the contact area, we assume that upon impact a spherical particle transforms to a half-ellipsoid with a height equal to the particle radius. Conserving the volume then leads to $A_{\mathrm {c}} = \pi d^2/2$. Substituting these relations into Eq. (1) and re-arranging it yields:

2 $$\frac{{\alpha \beta }}{6}\rho _p\left( {{\mathrm {d}}v_{{\mathrm {i,e}}}^5} \right)^{1/2} = \left( {\rho kC_p} \right)^{1/2} \times \left( {T_{\mathrm {m}} - T_0 + \frac{{H_{\mathrm {f}}}}{{C_{\mathrm {p}}}}} \right)$$

While the left-hand side of Eq. (2) is an impact-dependent term, the right-hand side can be regarded as the product of two material parameters, i.e, the thermal effusivity, $e_{{\mathrm {th}}} = ( {\rho kC_p} )^{1/2}$, and what we define as a melting index, $I_{{\mathrm {melt}}} = T_{\mathrm {m}} - T_0 + \frac{{H_{\mathrm {f}}}}{{C_p}}$. A fine estimation of the physical parameters *α* and *β* needs devoted case-by-case studies. Considering the uncertainty associated with the inelastic heat fraction, *β* can be treated as a calibration factor with a physical meaning. However, for a first-order approximation, we set a median value of *β* = 0.6 for the fraction of plastic work dissipated as heat in dynamic deformation^37,38^, and assume the initial energy is partitioned equally between the particle and the substrate (*α* = 0.5), especially for the present case where the particle and substrate materials are matched. Thus, we propose the threshold impact velocity that marks the onset of erosion should be approximately given by:

3 $$v_{{\mathrm {i,e}}} = \left( {\frac{{20e_{{\mathrm {th}}}I_{{\mathrm {melt}}}}}{{\rho _{\mathrm {p}}\sqrt d }}} \right)^{2/5}$$

While this analysis is a simple energy-balance with many approximations, Eq. (3) is nontrivial and presents an opportunity to test the hypothesis that melting controls erosion. Interestingly, a more physically based thermomechanical argument developed in Supplementary Note 1 implicitly supports the notion that erosion velocity is related to *e*_th_ × *I*_melt_. However, we highlight that neither in the energy-balance approach here nor in the thermomechanical approach developed in Supplementary Note 1, have we considered material strength effects. Strength effects are expected to be non-negligible when the deformation is not purely hydrodynamic. Thus, in what follows we limit ourselves to situations where hydrodynamic conditions would prevail, i.e., for conditions with relatively higher velocities, smaller particles, and softer metals.

**Fig. 4:**
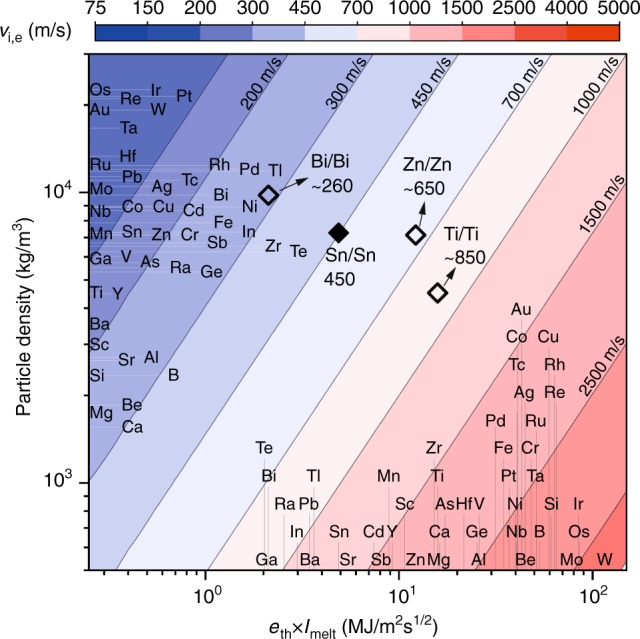
Melt-driven erosion map. Impact velocity at which melt-driven erosion is triggered for different combinations of particle/substrate materials. Particle material is populated on the *y*-axis. Material of interest in which erosion occurs (primarily substrate in the present context) is located on the *x*-axis. The intersecting point on the map determines the erosion velocity. The black diamond is the real-time measurement of tin erosion velocity with 4% uncertainty. The white diamonds are approximations of erosion velocity based on a limited number of site-specific impact experiments, and thus involve higher uncertainties

Figure 4 shows a map constructed based on Eq. (3) for 10-μm particles, in which we suggest the use of particle properties on the *y*-axis and substrate properties on the *x*-axis. By fixing particle diameter for this presentation, we are left with density on the *y*-axis to locate the particle material. The *x*-axis is the product of effusivity and the melting index, and can be used to locate the substrate material, when material loss from the substrate is concerned. Thus, for a given particle (horizontal line) and substrate (vertical line) there is an intersection position that is associated with a critical erosion velocity. The range of erosion velocities is represented by a series of contours in the present map.

We superimpose our in situ measurement of erosion velocity for tin particles impacting a tin substrate onto the map with a black diamond, and note a good agreement with the experimental critical erosion velocity of ~450 m/s lying on the correct theoretical contour line of the same velocity. To further confirm the versatility of the map, we conducted a limited number of site-specific impact experiments with zinc particles impacting zinc, bismuth particles impacting bismuth, and titanium particles impacting titanium (see Supplementary Figures 2–4). We estimate the lower bound erosion velocity based on the post-mortem observations of localized melting in the Supplementary Figures 2–4. All these cases are superimposed onto the map with white diamonds next to which the experimental erosion velocities are reported. Overall, we observe good agreement between the predictions of the map and the experimental measurements for different material pairs. This is a demonstration of the strength of the simple, but mechanistic, Eq. (3), and the introduction of *e*_th_ *×* *I*_melt_ as a material index to rank substrate materials can help advance materials design against erosion.

While our results are in good agreement with available estimations of erosion velocity^39^ for tin and zinc, both our experiment and theory predict erosion in titanium initiating at lower impact velocity than estimated previously. What is more, we do not see a major constraint in using Eq. (3) to predict erosion at larger scales. For example, measurements of mass gain after impacts of a 20-mm copper ball on a steel substrate show^39^ erosion at ~1000 m/s, and the emergence of material loss between ~350 and ~600 m/s. Eq. (3) predicts ~245 m/s to be the threshold velocity for melting for the same size copper ball impacting copper, which we interpret as being associated with the emergence of material loss. Underestimation of the erosion velocity by Eq. (3) compared to such macroscale experimental measurements can be attributed to two factors. First, the substrate in those experiments^39^ was much harder than copper while Eq. (3) predicts the velocity at which erosion would have emerged in a copper substrate. Second, at larger scales where the impact is more adiabatic, thermal conduction may be less significant in dictating the affected volume.

As a final note, and a direction for future studies, we highlight that as the impact velocity is increased, the erosion regime does not necessarily follow the bonding regime for all materials. For example, for bismuth impacts on bismuth (Supplementary Figure 2) we noted that rebound directly gives way to erosion, with no appreciable solid-state bonding in between. This can be justified by the fact that the two phenomena of bonding and erosion are governed by different mechanisms. Bonding is associated with solid-state hydrodynamic jetting^4,16^ whereas melt-driven erosion is governed by thermal properties of materials, as suggested by Eq. (3). Therefore, while Eq. (3) is the lower bound for melt-driven erosion, it can also be regarded as an upper bound velocity if an ideal impact-induced solid state bonding is sought.

To summarize, we have presented nanosecond and microscale in situ observations that span a broad range of possible regimes in matched materials impact. By extending the current understanding of microparticle impact from rebound and bonding to now include direct observations of erosion, we have provided the mechanistic insight that for normal-incidence microparticle impacts, such erosion in ductile materials is melt-driven. Simple mechanistic frameworks considering the conversion of impact kinetic energy into heat adequately capture the impact velocity at the onset of erosion. It also offers a material parameter that should prove useful for the design of materials to withstand erosive impacts, or for the design of additive manufacturing processes that rely on impact-bonding where the erosion regime must be avoided.

## Methods

### Materials and sample preparation

Tin, zinc, and bismuth powder particles with nominal particle sizes of -325 mesh, 6–9 μm, -100 mesh, as well as a tin plate with 3.2 mm thickness and a bismuth rod with 11 mm diameter were purchased from Alfa Aesar (Ward Hill, USA). A batch of titanium powder particles with nominal particle size of -325 mesh was purchased from AP&C (Quebec, Canada). A titanium plate and a zinc plate with 3.175 mm thickness were purchased from OnlineMetals (Seattle, USA). Supplementary Figure 1 shows the shape and the morphology of the powder particles. We used a diamond blade on a precision cutter to extract 12 × 12 mm plates for use as the targets for the impact experiments. Each target surface was ground and polished to nominally 0.04 µm mirror finish prior to the impact experiments. While impact of tin, zinc, and titanium particles is technologically relevant to cold spray coating/additive manufacturing^14,15,40–42^, bismuth enables us to explore impact-induced erosion of a metal with extremely low *e*×*I*_melt_.

### Single particle impact experiments

Single particle impact experiments were conducted using an in-house-designed all-optical microballistic platform. An intense laser pulse (pulsed Nd:YAG, 10-ns duration, 532-nm wavelength, pulse energy up to 60 mJ) is focused onto a launching assembly that consists of a glass substrate (210-μm thickness), a gold layer (60-nm thickness), and a polyurea film (30-μm thickness) on top of which metallic microparticles are dispersed. Following laser ablation of the gold film and rapid expansion of the polyurea film, the microparticles are accelerated toward the target. The particle speed is controlled by adjusting the laser energy (from 2 to 60 mJ). The laser pulse is focused into a 50-μm diameter spot size on the gold film using a 30-mm focal length lens. The distance between the launching pad and the target is typically 1 mm. The impact events are captured in real time using a μs laser pulse (10 μs duration, 640 nm wavelength) for illumination and a high-speed camera (SIMX 16, Specialized Imaging) that can acquire 16 frame videos with a rate up to 10^9^ fps and nanosecond time resolution. More details can be found elsewhere^4,5,22,23^.

During the impact experiments both particles and substrates are exposed to air. Thus, although we have not characterized surface oxide explicitly, we expect to have a typical native oxide of a few nm being present on both surfaces.

Impact and rebound velocities are determined by measuring the distance traveled by the particles in a few snapshots divided by the total interframe time between those snapshots. Particle velocities are extracted with an uncertainty of 4% taking into account the uncertainty in particle localization (±3 pixels corresponding to 1 μm) and in timing (±1 ns). More details on the uncertainties can be found elsewhere^21^. For each impact, the particle diameter was extracted from the image sequence. The average diameter for tin particles used in the impact experiments is 10 ± 1 µm. The particle size for each particle used in the site-specific impact experiments with bismuth, zinc, and titanium is reported in the captions of Supplementary Figures 2–4. Post-mortem observations of the impact residue were performed with Zeiss Merlin high-resolution scanning electron microscope. We conducted surface profilometry using a 3D laser scanning confocal microscope (VK-X200 series, Keyence), and measured the crater and pileup volumes accordingly.

## Electronic supplementary material

Supplementary Information

Description of Additional Supplementary Files

Supplementary Movie 1

Supplementary Movie 2

Supplementary Movie 3

Supplementary Movie 4

## Data Availability

The authors declare that the data supporting the findings of this study are available within the paper and its supplementary information file.
