# Supplementary material for: Melt-driven erosion in microparticle impact
Source: Nat Commun. 2018 Nov 29;9:5077. doi: 10.1038/s41467-018-07509-y (PMC6265329; doi:10.1038/s41467-018-07509-y)
Supplement: Supplementary file 1 — Supplementary Information [file 41467_2018_7509_MOESM1_ESM.pdf]

Melt-Driven Erosion in Microparticle Impact

Hassani-Gangaraj et al.

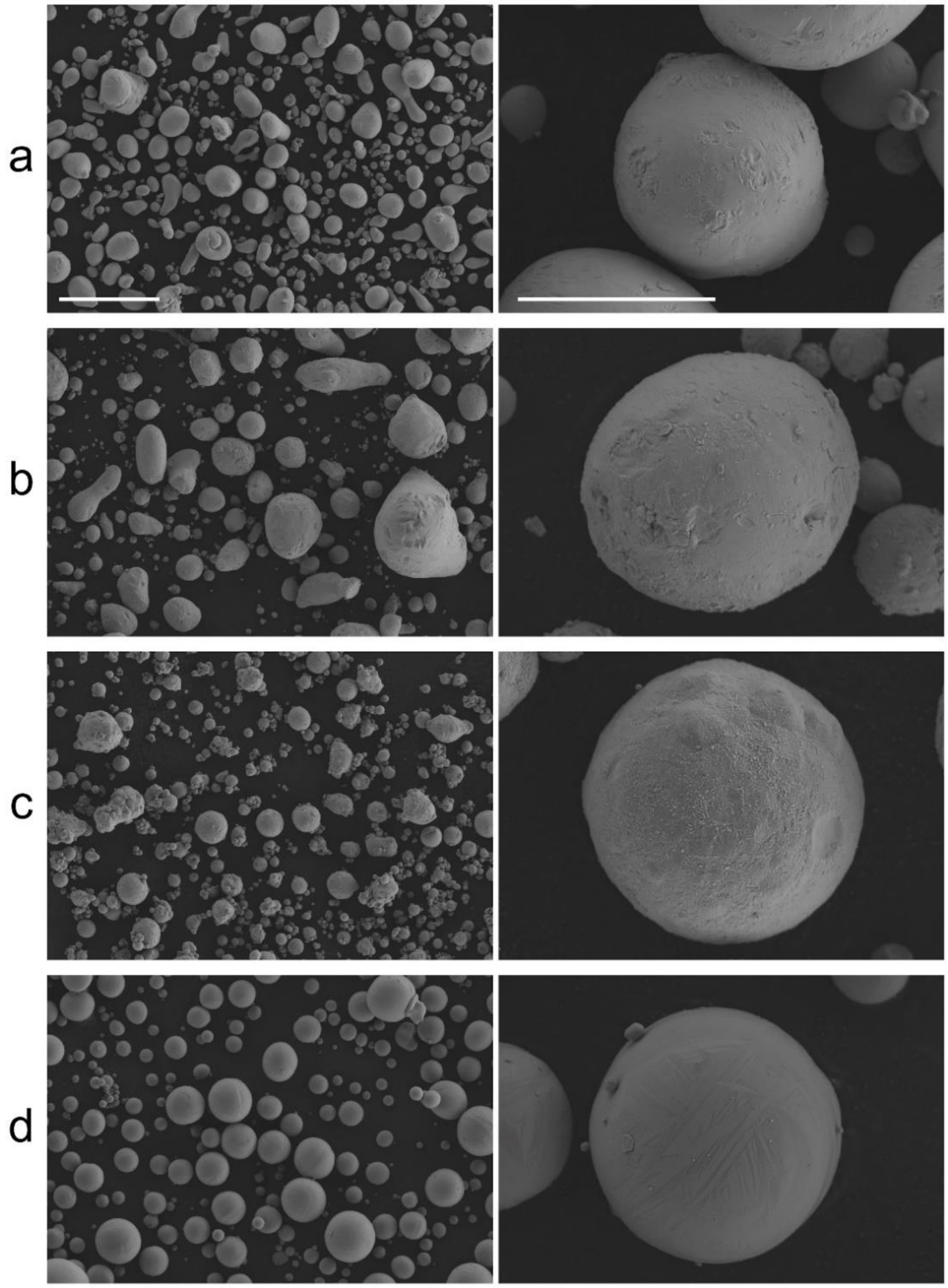

Supplementary Figure 1 | Powder Particles Before Impact. Low magnification (left) and high magnification (right) scanning electron micrographs of (a) tin, (b) bismuth, (c) zinc, and (d) titanium powder particles before impact. The scale bars are 50 µm for the images in the left and 10 µm for the images in the right.

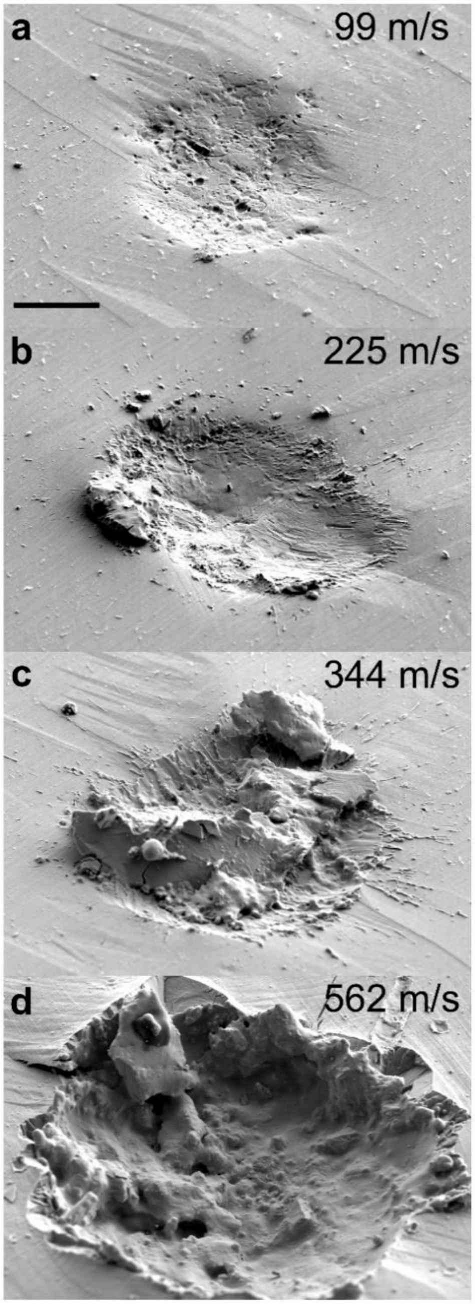

Supplementary Figure 2 | Site-specific experiments with Bi particles impacting Bi. Scanning electron micrographs of the impact areas after (a) 20-μm-, (b) 17-μm-, (c) 22-μm-, (d) 20-μm-bismuth particles impacted bismuth substrate at (a) 99, (b) 225, (c) 344, and (d) 562 m/s velocities. We estimate the velocity for the onset of melting for 20-μm Bi particles to be ~225 m/s. To superimpose this estimation on the map of Fig.3 in the text—that is constructed for 10-μm particles—we have adjusted this estimation using the size dependency in Eq. 3 in the text, i.e, 225 m/s $\times((20\ \mu m/10\ \mu m)^{1/2})^{2/5} \approx 260$ m/s. The scale bar is 5 μm.

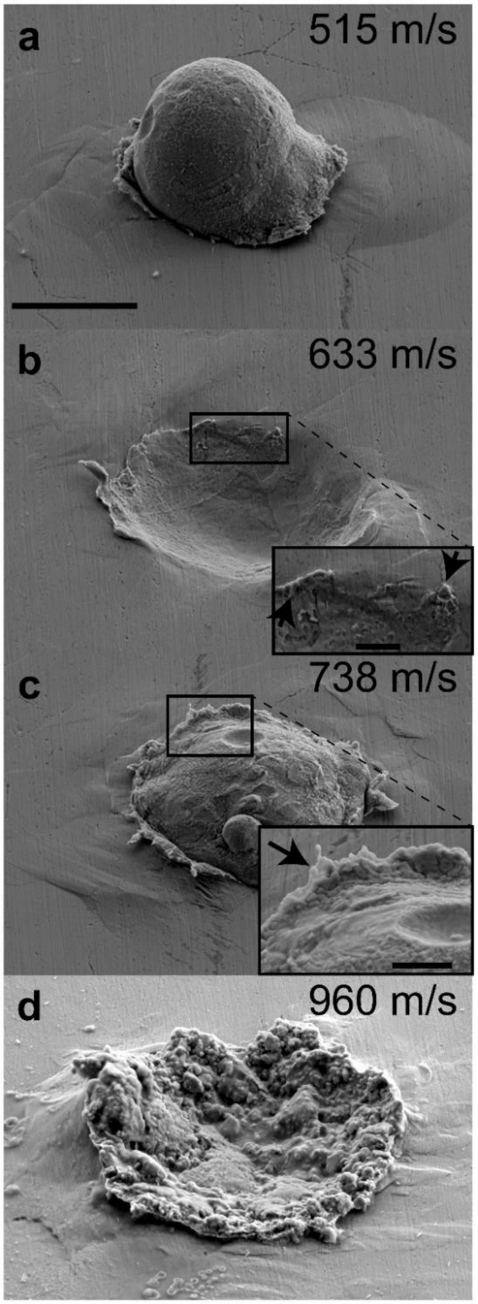

Supplementary Figure 3 | Site-specific experiments with Zn particles impacting Zn. Scanning electron micrographs of the impact areas after (a) 8-µm-, (b) 10-µm-, (c) 8-µm-, (d) 8-µm-zinc particles impacted zinc substrate at (a) 515, (b) 633, (c) 738, and (d) 960 m/s velocities. Shown with the arrows are signatures of localize melting. To superimpose Zn data point onto the map of Fig. 3 in the text, we estimate the velocity for the onset of melting for ~10-µm zinc particles to be ~650 m/s. The scale bar is 5 µm. The scale bars in the insets are 1 µm.

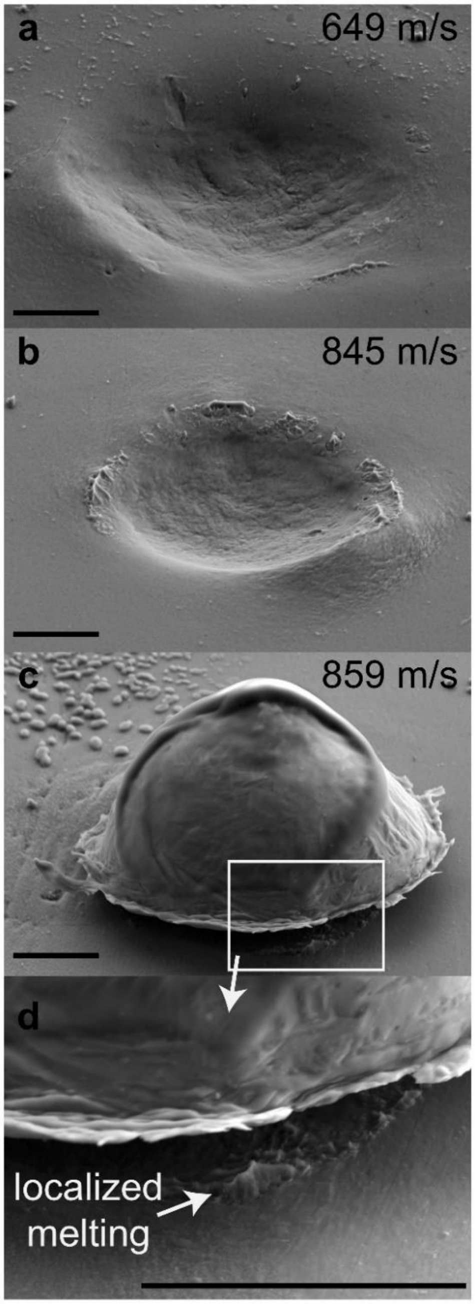

Supplementary Figure 4 | Site-specific experiments with Ti particles impacting Ti. Scanning electron micrographs of the impact areas after (a) 15-µm-, (b) 12-µm-, (c) 11-µm-titanium particles impacted titanium substrate at (a) 649, (b) 845, (c) 859 m/s velocities. To superimpose Ti data point onto the map of Fig. 3 in the text, we estimate the velocity for the onset of melting for ~10-µm titanium particles to be ~850 m/s. Zoomed-in images of the interface, shows (d) gap and signatures of localized melting. All scale bars are 5 µm.

Supplementary Note 1: Theoretical Framework

In the manuscript, we develop an energy-balance-based approach to estimate the conditions under which impact melting can occur. As an initial attempt to improve upon that simple approach, here we extend the theory with thermomechanical considerations, inspired by the framework developed by Schwarz and Koch[1]. Specifically, we aim to determine an upper bound estimate of temperature rise at low impact velocities in order to predict the threshold impact velocity for melting. We assume that particle and substrate, hereinafter taken to be the same material, are deformed by an effective stress, σ_{eff}, developed by impact. While in reality deformation is distributed inhomogeneously across the particle and substrate, here we idealize it by considering it to occur at the interface. Assuming that material at the interface moves at an effective velocity, v_{eff} , the energy flux, F, dissipated at the interface can be calculated by

$$F = \sigma_{eff}(v_i)v_{eff} \qquad (1)$$

where we take the effective stress to be itself a function of impact velocity. For a first-order approximation we assume that the effective velocity is of the same order as the impact velocity, $v_{eff} \approx v_i$. The energy flux is dissipated by conduction one-dimensionally away from the interface, half of the heat escaping to either side. The temperature increase, ΔT, at the surface of a semi-infinite solid subject to heat flux $F/2$ for time Δt is given by[2]:

$$\Delta T = F\left(\frac{\Delta t}{\pi k \rho_p C_p}\right)^{1/2} \qquad (2)$$

where k is thermal conductivity and c_p is specific heat. Assuming the process lasts for $\Delta t = d/v_i$ and introducing thermal effusivity $e_{th} = \sqrt{\rho k C_p}$ we can re-write equation S2:

$$\Delta T = \frac{\sigma_{eff}(v_i)}{e}\left(\frac{d v_i}{\pi}\right)^{1/2} \qquad (3)$$

At the melting threshold, we can substitute temperature increase, ΔT, with the “melting index” defined in the manuscript as $I_{melt} = T_m - T_0 + \frac{H_f}{C_p}$. At the melting threshold, we can also substitute impact velocity v_i with the erosion velocity $v_{i,e}$. Re-arranging Supplementary Equation 3 then yields:

$$v_{i,e} = \frac{\pi}{d}\left(\frac{e_{th} I_{melt}}{\sigma_{eff}(v_i)}\right)^2 \tag{4}$$

Supplementary Equation 4 is not explicit in terms of erosion velocity. However, it demonstrates the importance of $e_{th} \times I_{melt}$, in accord with the Equation 3 in the text, although the two are derived from different routes—a thermomechanical approach for Supplementary Equation 4 and an energy-balance-based approach for Equation 3. Interestingly, Supplementary Equation 4 would have the same functional form as Equation 3 in the text if the impact-induced effective stress in the present simplified model of the deformation were proportional to ρv_i^2, that is to say, if the deformation were hydrodynamic and material strength effects therefore neglected. If conditions for hydrodynamic deformation are not met, a material would behave more like a solid upon impact than a liquid. In such cases, stresses that are sustained during the deformation should be on the order of material’s strength. Neither Equation 3 in the text, nor Supplementary Equation 4 account for the material’s strength explicitly, and would need modifications to capture the actual dynamics of melting/erosion in high-velocity impact under conditions that do not achieve hydrodynamic conditions.

Supplementary References

1. Schwarz, R. B. & Koch, C. C. Formation of amorphous alloys by the mechanical alloying of crystalline powders of pure metals and powders of intermetallics. *Appl. Phys. Lett.* **49,** 146–148 (1986).

2. Carslaw, H. S. & Jaeger, J. C. *Conduction of Heat in Solids*. (Clarendon Press, 1986).
