# Supplementary material for: Melt-driven erosion in microparticle impact
Source: Nat Commun. 2018 Nov 29;9:5077. doi: 10.1038/s41467-018-07509-y (PMC6265329; doi:10.1038/s41467-018-07509-y)
Supplement: Supplementary file 2 — Description of Additional Supplementary Files [file 41467_2018_7509_MOESM2_ESM.pdf]

Description of Additional Supplementary Files

File Name: Supplementary Movie 1
Description: Impact of a 9-µm tin particle on a tin substrate at 336 m/s (± 4%) velocity. The 16-frame video has a 400 × 300 µm field of view and an interframe time 150 ns of for a total duration of 2250 ns duration. The particle rebounds after being flattened upon impact.

File Name: Supplementary Movie 2
Description: Impact of a 9-µm tin particle on a tin substrate at 374 m/s (± 4%) velocity. The 16-frame video has a 400 × 300 µm field of view and an interframe time 150 ns of for a total duration of 2250 ns duration. The particle does not rebound, but instead bonds to the substrate.

File Name: Supplementary Movie 3
Description: Impact of a 10-µm tin particle on a tin substrate at 678 m/s (± 4%) velocity and its rebound. The 16-frame video has a 400 × 300 µm field of view and an interframe time 75 ns of for a total duration of 1125 ns duration. A quick splash with succeeding fragmentations contribute to material loss.

File Name: Supplementary Movie 4
Description: Impact of a 10-µm tin particle on a tin substrate at 1067 m/s (± 4%) velocity and its rebound. The 16-frame video has a 400 × 300 µm field of view and an interframe time 50 ns of for a total duration of 750 ns duration. Material is almost entirely lost by splash.
